# Association between Body Mass Index and Prognosis of Colorectal Cancer: A Meta-Analysis of Prospective Cohort Studies

**DOI:** 10.1371/journal.pone.0120706

**Published:** 2015-03-26

**Authors:** Junga Lee, Jeffrey A. Meyerhardt, Edward Giovannucci, Justin Y. Jeon

**Affiliations:** 1 Department of Sport and Leisure Studies, Yonsei University, Seoul, South Korea; 2 Exercise Medicine Center for Diabetes and Cancer Patients, Yonsei University, Seoul, Korea; 3 Dana Farber Cancer Institute, Harvard Medical School, Boston, MA, United States of America; 4 Departments of Nutrition and Epidemiology, Harvard School of Public Health, Boston, MA, United States of America; Institute of Pathogen Biology, CHINA

## Abstract

Studies have reported conflicting results on the association between body mass index (BMI) and prognosis of colorectal cancer. Therefore, we have conducted a meta-analysis of prospective studies, which examined the association of pre- and post-diagnostic BMI with colorectal cancer-specific mortality and all-cause mortality in patients with colorectal cancer. We searched Medline and EMBASE database published between 1970 and September 2014. A total of 508 articles were identified, of which 16 prospective cohort studies were included for the current meta-analysis. The analysis included 58,917 patients who were followed up over a period ranging from 4.9 to 20 years (median: 9.9 years). We found that being underweight before cancer diagnosis was associated with increased all-cause mortality (Relative risk [RR]: 1.63, 95% CI: 1.18–2.23, *p* < 0.01) and being obese (BMI ≥ 30 kg/m^2^) before cancer diagnosis was associated with increased colorectal cancer-specific mortality (RR: 1.22, 95% CI: 1.003–1.35, *p* < 0.01) and all-cause mortality (RR: 1.25, 95% CI: 1.14–1.36, *p* < 0.01). On the other hand, being underweight (RR: 1.33, 95% CI: 1.20–1.47, *p* < 0.01), obese (RR: 1.08, 95% CI: 1.03–1.3, *p* < 0.01), and class II/III obese (BMI ≥ 35 kg/m^2^; RR: 1.13, 95% CI: 1.04–1.23, *p* < 0.01) after diagnosis were associated with significantly increased all-cause mortality. Being obese prior to diagnosis of colorectal cancer was associated with increased colorectal cancer-specific mortality and all-cause mortality, whereas being obese after diagnosis was associated with increased all-cause mortality. The associations with being underweight may reflect reverse causation. Maintaining a healthy body weight should be discussed with colorectal cancer survivors.

## Introduction

Each year, over 1.2 million new cases of colorectal cancer are reported, resulting in 600,000 deaths. Colorectal cancer has become the third most common cancer in the world, making it the fourth leading cause of cancer mortality [1]. One of the primary risk factors for colorectal cancer is obesity, a condition typically assessed using a scale known as the body mass index (BMI) [2–4]. A recent meta-analysis that systematically reviewed 23 studies (168,201 participants) reported that participants with a BMI greater than 25 kg/m^2^ had a 24% increased prevalence of colorectal adenomas [5]. Another recent meta-analysis with 41 studies also found that obesity was associated with a 33% increased risk of colorectal cancer among 8,115,689 participants [6]. The association between BMI and prognosis of colorectal cancer is less clear.

Understanding the association between BMI and the prognosis of colorectal cancer is highly important to provide body weight guidelines for colorectal cancer patients. Studies have clearly identified that being underweight is associated with increased risk of death, probably due to cancer progression-associated weight loss [7–10]. However, the association between being overweight and the risk of mortality is less clear. Baade et al. [7] and Kuiper et al. [11] reported that colorectal cancer patients who were overweight had 25% and 55% improved colorectal cancer-specific mortality, respectively. On the other hand, other studies reported no difference in the risk of mortality among overweight compared with normal weight colorectal cancer patients [8,10,12]. Inconsistent findings also been observed among studies which examined the association between being obese and the prognosis of colorectal cancer; some reported increased mortality [12,13] while others reported reduced mortality among obese colorectal cancer patients [7]. Due to these mixed findings, it is difficult for oncologists to provide evidence-based guidelines for ideal body weight for colorectal cancer patients. Because these inconsistencies could be due to small sample sizes and time of BMI measurement (before or after diagnosis), a meta-analysis is needed.

Recently, Parkin et al. [35] comprehensively performed and reported systemic review analysis to study the impact of body adiposity on prognosis of colorectal cancer. Although being underweight is one of the important prognostic factors for colorectal cancer patients, Parkin et al. [35] did not study the association between being underweight and prognosis of colorectal cancer patients. Therefore, we performed a meta-analysis of prospective cohort studies to identify the association between BMI (before and after diagnosis) including being underweight and the prognosis of colorectal cancer patients, including colorectal cancer-specific mortality and all-cause mortality.

## Methodology

### Search Strategy

This meta-analysis study followed the guidelines provided by the preferred reporting items for systemic review and meta-analyses [2]. We conducted an extensive search for articles that studied the association between BMI and colorectal cancer mortality. The electronic databases MEDLINE and EMBASE were used to search eligible studies published in English language peer-reviewed journals from January 1970 to September 2014.

The search terms (used in various combinations) were ‘body mass index (BMI)’ ‘colorectal cancer’, ‘colon cancer’, ‘rectal cancer’ ‘mortality’, ‘survival’, ‘over all survival, ‘cancer-specific survival’, ‘disease free survival’, and ‘prognosis free survival’. Also, we completed a manual search of references cited in the selected articles and review articles to explore for any further relevant studies. All potentially relevant studies were archived in an Endnote X6 database.

### Eligibility Criteria

The selected articles were independently screened in an unblinded standardized manner by two authors (JL and JYJ). Any discrepancies regarding eligibility for study selection were re-evaluated by further discussion involving all four authors (JL, JYJ, JAM and EG) in order to reach consensus. The studies were assessed for eligibility using both inclusion and exclusion criteria. The inclusion criteria required that studies had a prospective study design and contained data that addressed all-cause mortality and colorectal cancer-specific mortality, with data on pre-diagnosis BMI or post-diagnosis BMI. Two case-control studies, which were converted to a survival cohort with recalled body weight, were included in this analysis [7,12]. In the case of multiple published reports on the same study population, only the study with the longest follow-up was included.

### Data Extraction

This analysis followed the Meta-analysis of Observational Studies in Epidemiology (MOOSE) guidelines for meta-analysis of observational studies [14]. To ensure compliance with MOOSE guidelines, all selected studies were double checked by two authors (JL and JYJ). Any discrepancies with the extracted data led to further discussion among the four authors in order to reach consensus (JL, JYJ, JAM, and EG). Data extraction in this meta-analysis recorded the following elements: last name of the first author, publication year, country in which the study was performed, sample size, number of deaths, age at baseline, gender, assessment method for the BMI measurement (self-reported vs. measured), adjustment factors, relative risks (RRs) with corresponding 95% confidence intervals (CI), and duration of follow-up.

The Newcastle-Ottawa Scale (NOS) procedure was used to assess the quality of the study [4]. The NOS procedure was selected as it provides an easy and convenient tool for the quality assessment of non-randomized studies to be used in a systematic review. This assessment examined the following items: clarity of BMI measurement (pre-diagnosis and post-diagnosis), adjustment for intermediate factors (e.g., age, stage and tumor differentiation), duration of follow-up, study endpoints (colorectal cancer-specific mortality and overall mortality), representativeness of the exposed cohort, and adequacy of follow-up of cohorts (Tables 1 & 2).

**Table 1:** Prospective cohort studies of pre-diagnosis BMI (kg/m^2^) and survival outcomes in colorectal cancer (CRC) patients.

First author (year), name of study, country	Follow-up period(years)	Sample characteristics (gender, age, location of tumor, number of events)	Measure of BMI	RR (95% CI)		Adjustment factors
Doria-Rose (2006) [22], Wisconsin Cancer Reporting System, U.S.A	9.4 years	633 CRC cases (females, post-menopausal) aged 38–74 years, 280 deaths and 147 CRC	5 year before interview (1990–1992)			Age, stage, PMH use, and smoking
				**CRC-specific mortality**		
		Stage I-III		<20.0	1.60(0.88–2.92)	
				20.0–24.9	1	
				25.0–29.9	1.30(0.89–1.89)	
				≥30	1.50(0.88–2.55)	
				**All-cause mortality**		
				<20.0	1.50(0.92–2.45)	
				20.0–24.9	1	
				25.0–29.9	1.20(0.90–1.60)	
				≥30	1.50(0.88–2.55)	
Prizment (2010) [15], Iowa Women’s Health study, U.S.A.	20 years	1,096 females CC aged 56–89 years 493 deaths and 239 CC deaths	Self-reported BMI at baseline in 1986			Stage, age, education, smoking, first course treatment surgery, chemotherapy, and radiation
				**CRC-specific mortality**		
		Stage I-III		<18.5	1.84(0.84–4.03)	
				18.5–24.9	1	
				25.0–29.9	1.18(0.87–1.52)	
				≥30	1.35(1.00–1.82)	
				**All-cause mortality**		
				<18.5	1.89(1.01–3.53)	
				18.5–24.9	1	
				25.0–29.9	1.12(0.89–1.41)	
				≥30	1.45(1.14–1.85)	
Kuiper (2012) [11], Women’s Health Initiative,U.S.A.	11.9 years	1,339 females aged 57–72 years, 1,082 CC, 257 RC, 265 deaths, 171 CRC-specific deaths	5.8 years before diagnosis (1993–1998)			Age, study arm, time from diagnosis to measurement, pre-diagnostic BMI, tumor stage, ethnicity, education, alcohol, smoking, and hormone therapy use
				**CRC-specific mortality**		
		Stage I-IV		18.5–24.9	1	
				25.0–29.9	0.77(0.52–1.13)	
				≥30	1.17 (0.80–1.72)	
				**All-cause mortality**		
				18.5–24.9	1	
				25.0–29.9	0.90(0.66–1.23)	
				≥30	1.19(0.88–1.62)	
Pelser (2014) [18], NIH-AARP Diet and Health Study, U.S.A.	5 years	4,213 CC and 1,514 RC aged 68.4–70.5 years, 1,273 deaths (856 CRC deaths, 125 other cancers, 108 Cardiovascular disease, 184 other cause)	Self-reported BMI at baseline (1995–1996)			Age, lag time, gender, education, family history of colon cancer, cancer stage, and first course of treatment (surgery, radiation, chemotherapy)
				**CRC-specific mortality among colon cancer cases**		
		Stage I-IV		18.5–24.9	1	
				25.0–29.9	0.97(0.82–1.15)	
				≥30	1.15 (0.96–1.39)	
				**All-cause mortality**		
				18.5–24.9	1	
				25.0–29.9	1.02(0.88–1.17)	
				≥30	1.19(1.02–1.39)	
				**CRC-specific mortality**		
				18.5–24.9	1	
				25.0–29.9	0.92(0.70–1.22)	
				≥30	1.04 (0.75–1.44)	
				**All-cause mortality**		
				18.5–24.9	1	
				25.0–29.9	0.85(0.68–1.07)	
				≥30	1.00(0.77–1.30)	
Campbell (2012) [16], Cancer Prevention Study-II Nutrition Cohort, U.S.A.	16 years	2,303 (1,291 males & 1,012 females) aged younger than 65 to older than 80 years, 380 CRC-specific deaths, 851 All-cause deaths, 153 Cardiovascular disease specific deaths	7 years before diagnosis (1992–1993) Self-reported BMI			Age, sex, smoking status, BMI, physical activity, red meat intake, tumor stage, and education
				**CRC-specific mortality**		
		Stage II-III		Female		
				<18.5	0.83(0.25, 2.76)	
				18.5–24.9	1	
				25.0–29.9	1.19(0.80, 1.78)	
				≥30	1.52(0.96, 2.41/	
				Male		
				<18.5	0	
				18.5–24.9	1	
				25.0–29.9	1.06(0.77, 1.48)	
				≥30	1.31(0.88, 1.95)	
				Both		
				<18.5	0.67(0.21, 2.12)	
				18.5–24.9	1	
				25.0–29.9	1.09(0.85, 1.40)	
				≥30	1.35(1.01, 1.80)	
				**All-cause mortality**		
				Female		
				<18.5	1.74(0.85, 3.58)	
				18.5–24.9	1	
				25.0–29.9	1.22(0.95, 1.63)	
				≥30	1.42(1.01, 2.00)	
				Male		
				<18.5	1.40(0.55, 3.56)	
				18.5–24.9	1	
				25.0–29.9	0.97(0.79, 1.19)	
				≥30	1.21 (0.94, 1.57)	
				Both		
				<18.5	1.53(0.88, 2.66)	
				18.5–24.9	1	
				25.0–29.9	1.06(0.90, 1.25)	
				≥30	1.30(1.06, 1.58)	
Fedirko (2014) [35], European Prospective investigation into Cancer and Nutrition (EPIC), European	4 years	3,924 CRC case 1, 309 deaths (1,043 CRC deaths)	BMI at baseline (1992–1998)			Sex, age at diagnosis, smoking status, year of diagnosis, CRC state, grade, tumor location, alcohol consumption, sex-specific categories of physical activity, and intake of fish and shellfish, and fruits and vegetables.
		Stage I-IV		**CRC-specific mortality**		
				< 25	1	
				25.0–29.9	1.06(0.87–1.28)	
				≥30	1.28 (1.00–1.63)	
				**All-cause mortality**		
				< 25	1	
				25.0–29.9	-	
				≥30	1.32(1.12–1.56)	

Table 1 is a summary of pre-diagnosis studies. This assessment examined the following items: clarity of BMI measurement before diagnosis, adjustment for intermediate factors (e.g., age, stage and tumor differentiation), duration of follow-up, study endpoints (colorectal cancer-specific mortality and overall mortality), representativeness of the exposed cohort, and adequacy of follow-up of cohorts.

Abbreviations: BMI, body mass index; RR, risk ratio; CI, confidence interval; CRC, colorectal cancer; RC, rectal cancer; CC, colon cancer; PMH, post-menopausal hormone

**Table 2:** Prospective cohort studies of post-diagnosis BMI (kg/m^2^) and survival outcomes in colorectal cancer patients.

First author (year), name of study, country	Follow-up period (years)	Sample characteristics (gender, age, disease stage, location of tumor, number of events)	Measure of BMI	RR (95% CI)		Adjustment factors
Meyerhardt (2003) [10], Intergroup Trial 0089, U.S.A.	9.4 years	3,759 CC (females & males; stage II & III) aged younger than 50 to older than 70 years	BMI measured on day 1 of chemotherapy (1988–1992)			Age, race, baseline, performance status, bowel obstruction, bowel perforation, Duke stage, presence of peritoneal implants, predominant macroscopic pathologic feature, and completion of chemotherapy
				**All-cause mortality**		
		Stage II-III		Female		
				<21	1.08(0.87–1.35)	
				21–24.9	1	
				25.0–27.49	1.18(0.94–1.49)	
				27.5–29.9	1.23(0.95–1.60)	
				≥30	1.34(1.07–1.67)	
				Male		
				<21	1.33(1.05–1.67)	
				21–24.9	1	
				25.0–27.49	1.03(0.87–1.22)	
				27.5–29.9	0.96(0.78–1.17)	
				≥30		
				Both		
				<21	1.15(0.98–1.35)	
				21–24.9	1	
				25.0–27.49	1.10(0.95–1.26)	
				27.5–29.9	1.05(0.90–1.24)	
				≥30	1.11(0.96–1.29)	
Meyerhardt (2004) [8], National Cancer Insititue-0114, U.S.A.	9.9 years	1,688 females & males RC, Inclusion Age and Number of Deaths not Reported	BMI measured on day 1 of chemotherapy (1990–1992)			Age, sex, race, baseline performance status, bowel obstruction, extent of bowel wall invasion, and number of positive lymph nodes
				**All-cause mortality**		
		Stage II-III		Female		
				<20	1.29(0.87–1.91)	
				20–24.9	1	
				25.0–27.49	0.75(0.49–1.16)	
				27.5–29.9	0.89(0.61–1.33)	
				≥30	0.94(0.66–1.33)	
				Male		
				<20	1.62(1.08–2.43)	
				20–24.9	1	
				25.0–27.49	1.07(0.86–1.33)	
				27.5–29.9	0.99(0.79–1.25)	
				≥30	1.19(0.94–1.52)	
				Both		
				<20	1.43(1.08–1.89)	
				20–24.9	1	
				25.0–27.49	0.97(0.80–1.17)	
				27.5–29.9	0.95(0.78–1.15)	
				≥30	1.09(0.9–1.33)	
Dignam (2006) [12], National Surgical Adjuvant Breast and Bowel Project randomized trials, U.S.A.	11.2 years	4,288 females CC aged younger than 40 to older than 60 years, 1697 total deaths (1,159 CC deaths & 538 non-CC deaths)	BMI measured at diagnosis (1989–1994)			Treatment, age, sex, race, performance status, number of positive lymph nodes, and presence of bowel obstruction
				**All-cause mortality**		
		Stage I-III		<18.5	1.49(1.17–1.91)	
				18.5–24.9	1	
				25.0–29.9	1.02(0.91–1.14)	
				30–34.9	1.11(0.96–1.28)	
				≥35	1.28(1.04–1.57)	
Meyerhardt (2008) [9], CALGB 89803, U.S.A.	5.3 years	1,053 females & males CC aged 55–64 years, 261 death	Self-reported BMI at 6 month after adjuvant chemotherapy (1999–2001)			Sex, age, depth of invasion through bowel wall, number of positive lymph node, presence of clinical perforation at time of surgery, presence of bowel obstruction, baseline CEA, grade of tumor differentiation, baseline performance status, treatment arm, weight change between first and second questionnaire, BMI at the time or second questionnaire, and time between study entry and completion of second questionnaire
		Stage III		**All-cause mortality**		
				<21	1.07(0.61–1.87)	
				21–24.9	1	
				25.0–27.49	0.72(0.50–1.03)	
				27.5–29.9	0.90(0.61–1.34)	
				≥30	0.87(0.54–1.42)	
Sinicrope (2010) [23], National Cancer Institute and conducted by Mayo Clinic/North Central Cancer Treatment Group and the Southwest Oncology group, U.S.A.	8 years	4,381 females & males aged average 60.4 years, 1,833 death	BMI measured at study entry Inclusion years not reported			Age, stage, treatment and gender
				**All-cause mortality**		
		Stage II-III		Female		
				<20	1.32(1.05–1.67)	
				20–24.9	1	
				25.0–27.49	1.18(0.94–1.49)	
				27.5–29.9	1.24(1.01–1.53)	
				≥30	1.11(0.84–1.45)	
				Male		
				<20	1.14(0.81–1.61)	
				20–24.9	1	
				25.0–27.49	0.82(0.71–0.95)	
				27.5–29.9	0.94(0.78–1.15)	
				≥30	1.35(1.02–1.79)	
				Both		
				<20	1.24(1.03–1.5)	
				20–24.9	1	
				25.0–27.49	0.90(0.8–1.00)	
				27.5–29.9	1.07(0.93–1.23)	
				≥30	1.19(0.98–1.45)	
Baade (2011) [7], Queensland, Australia	4.9 years	1825 (1,089 males and 736 females) aged 20 to older than 70 years, 1163 colon cancer, 662 rectal cancer, 462 deaths, 345 CRC-specific deaths	Self-reported BMI at 5 months after diagnosis (2003–2004)			Age, sex, stage at diagnosis, smoking, site of tumor, and treatment (surgery only vs. surgery and adjuvant therapy)
				**CRC-specific mortality**		
		Stage I-III		<18.5	1.74(0.85, 3.58)	
				18.5–24.9	1	
				25.0–29.9	0.75 (0.59–0.97)	
				≥30	1.34 (0.70–2.58)	
				**All-cause mortality**		
				<18.5	2.29 (1.47–3.59)	
				18.5–24.9	1	
				25.0–29.9	0.75 (0.61–0.94)	
				≥30	0.94 (0.51–1.74)	
Chin (2012) [17], China	5 years	2,765 females & males aged average 61.4 ± 13.9 years,	BMI measured after diagnosis (1995–2003)			Tumor, nodes, and metastasis stage, age, gender, comorbidities, carcinoembryonic antigen, hemoglobin, albumin, operative timing, postoperative morbidity, tumor location, histologic type, and histologic grade
		Stage I-III		**CRC-specific mortality**		
				Female		
				<18.5	1.16(0.75–1.82)	
				18.5–24.9	1	
				25.0–29.9	0.96(0.60–1.43)	
				≥30	1.11(0.84–1.43)	
				Male		
				<18.5	1.46(0.84–2.52)	
				18.5–24.9	1	
				25.0–29.9	0.96(0.69–1.32)	
				≥30	1.21(0.83–1.77)	
				Both		
				<18.5	1.33(0.94–1.87)	
				18.5–24.9	1	
				25.0–29.9	0.96(0.76–1.2)	
				≥30	1.06(0.80–1.41)	
				**All-cause mortality**		
				Female		
				<18.5	1.55(1.11–2.16)	
				18.5–24.9	1	
				25.0–29.9	0.95(0.71–1.27)	
				≥30	0.99(0.69–1.41)	
				Male		
				<18.5	1.55(1.03–2.35)	
				18.5–24.9	1	
				25.0–29.9	0.77(0.58–1.01)	
				≥30	0.91(0.66–1.25)	
				Both		
				<18.5	1.58(1.23–2.05)	
				18.5–24.9	1	
				25.0–29.9	0.84(0.65–1.09)	
				≥30		
Kuiper (2012) [11], Women’s Health Initiative, U.S.A.	11.8 years	676 females aged 57–72 years, 54 CRC-specific deaths, 101 All-cause deaths	BMI measured 0.8 years after diagnosis (1993–1998)			Age, study arm, time from diagnosis to measurement, pre-diagnostic BMI, tumor stage, ethnicity, education, alcohol, smoking, and hormone therapy use
				**CRC-specific mortality**		
				18.5.0–24.9	1	
		Stage I-IV		25.0–29.9	0.45(0.22–0.92)	
				≥30	0.95(0.49–1.85)	
				**All-cause mortality**		
				18.5.0–24.9	1	
				25.0–29.9	0.78(0.47–1.27)	
				≥30	1.09(0.65–1.83)	
Campbell (2012) [16], Cancer Prevention Study-II Nutrition Cohort, U.S.A.	6.8 years	1,957 (1,291 males & 1,012 females) aged younger than 65 to older than 80 years, 273 CRC-specific deaths, 683 All-cause deaths, 135 Cardiovascular disease specific deaths	Self-reported measured after diagnosis (1992–1993)			Age, sex, smoking status, BMI, physical activity, red meat intake, tumor stage, and education
				**CRC-specific mortality**		
		Stage I-III		Female		
				<18.5	0.39(0.12, 1.32)	
				18.5–24.9	1	
				25.0–29.9	0.81(0.50, 1.31)	
				≥30	1.09(0.60, 2.01)	
				Male		
				<18.5	2.48(0.55,11.3)	
				18.5–24.9	1	
				25.0–29.9	0.91(0.61, 1.34)	
				≥30	1.29 (0.82, 2.01)	
				Both		
				<18.5	0.64(0.25, 1.60)	
				18.5–24.9	1	
				25.0–29.9	0.87(0.65, 1.17)	
				≥30	1.14 (0.81, 1.60)	
				**All-cause mortality**		
				Female		
				<18.5	1.19(0.65, 2.18)	
				18.5–24.9	1	
				25.0–29.9	0.84(0.60, 1.16)	
				≥30	1.19 (0.79, 1.78)	
				Male		
				<18.5	2.78(1.29, 5.96)	
				18.5–24.9	1	
				25.0–29.9	0.82(0.66, 1.03)	
				≥30	0.89(0.67, 1.18)	
				Both		
				<18.5	1.30(0.82, 2.06)	
				18.5–24.9	1	
				25.0–29.9	0.83(0.70, 1.00)	
				≥30	0.93 (0.75, 1.17)	
Sinicrope (2013) [13], ACCENT Group database, U.S.A.	7.8 years	25.291 females & males aged 43–71 years CC	BMI measured at study entry Inclusion years not reported			Age, stage, treatment, and sex
				**All-cause mortality**		
		Stage I-III		Female		
				<20	1.12(1.00–1.25)	
				20–24.9	1	
				25.0–29.9	1.05(0.97–1.14)	
				30–34.9	1.10(0.99–1.23)	
				≥35	1.07(0.93–1.24)	
				Male		
				<20	1.39(1.21–1.60)	
				20–24.9	1	
				25.0–29.9	0.95(0.87–1.02)	
				30–34.9	1.10(1.99–1.2)	
				≥35	1.16(1.0–1.35)	
				Both		
				<20	1.21(1.11–1.32)	
				20–24.9	1	
				25.0–29.9	1.10(1.04–1.17)	
				30–34.9	1.10(1.02–1.18)	
				≥35	1.11(1.00–1.23)	
Boyle (2013) [20], The Western Australia Bowel Health Study, Australia	5.9 years	918 females & males aged 40–79 years CRC, 224 deaths (69 females &155 males)	BMI measured 1 year before study enrolment			Age, sex, socioeconomic status, tumor stage and diabetes, physical activity, body mass index and smoking
				**CRC-specific mortality**		
		Stage I-IV		Both (Stage IV)		
				<25	1	
				25.0–29.9	1.37(0.55, 3.42)	
				≥30	1.06 (0.31, 3.57)	
				Both (Stage I-III)		
				<25	1	
				25.0–29.9	1.53(0.98, 2.38)	
				≥30	1.49(0.92, 2.40)	
				**All-cause mortality**		
				Both (Stage IV)		
				<25	1	
				25.0–29.9	1.37(0.55, 3.42)	
				≥30	1.06 (0.31, 3.57)	
				Both (Stage I-III)		
				<25	1	
				25.0–29.9	1.33(0.90, 1.96)	
				≥30	1.34(0.88, 2.04)	
Schlesinger (2014) [21], PopGen Biobank, Germany	4 years	2,143 females & males 349 deaths	Self-reported measured after diagnosis			Age, sex, alcohol consumption, smoking status, tumor location, family history of CRC, metastases and other cancer
				**All-cause mortality**		
		Stage I-III if colon cancer		<18.5	1.65(0.79, 3.46)	
				18.5–24.9	1	
				25.0–29.9	0.80(0.62, 1.02)	
				≥30	0.84(0.62, 1.14)	

Table 2 is a summary of post-diagnosis studies. This assessment examined the following items: clarity of BMI measurement after diagnosis, adjustment for intermediate factors (e.g., age, stage and tumor differentiation), duration of follow-up, study endpoints (colorectal cancer-specific mortality and overall mortality), representativeness of the exposed cohort, and adequacy of follow-up of cohorts.

Abbreviations: BMI, body mass index; RR, risk ratio; CI, confidence interval; CRC, colorectal cancer; RC, rectal cancer; CC, colon cancer; PMH, post-menopausal hormone

### Statistical Analysis

Individual study RRs were directly extracted from the published reports, and inserted into the Comprehensive Meta-Analysis Version 1.25 software program that computed fixed and random-effect model parameters and 95% confidence intervals (CIs). Statistical heterogeneity across the sampled studies was tested using the Q statistic, and inconsistency was quantified by the *I* ^*2*^ statistic. When performing meta-analysis, fixed effect models were used when selected studies for meta-analysis were homogenous. On the other hand, random-effect models with forest plots were used for meta-analysis when selected studies were not homogeneous. In further sub-analysis, additional meta-analyses were performed for genders and site of diseases. To assess for publication bias, a visual inspection of the funnel plot was conducted to find the relationship between the study results and precision. Trim and fill analyses were used to test the potential influence that unpublished studies could have on the summary RR estimates. Statistical significance was tested using a p-value of < 0.05. All statistical analyses were performed using Comprehensive Meta-Analysis Version 1.25 software (Biostatic, Inc., Englewood, NJ, USA).

## Results

### Literature Search

**Fig 1:**
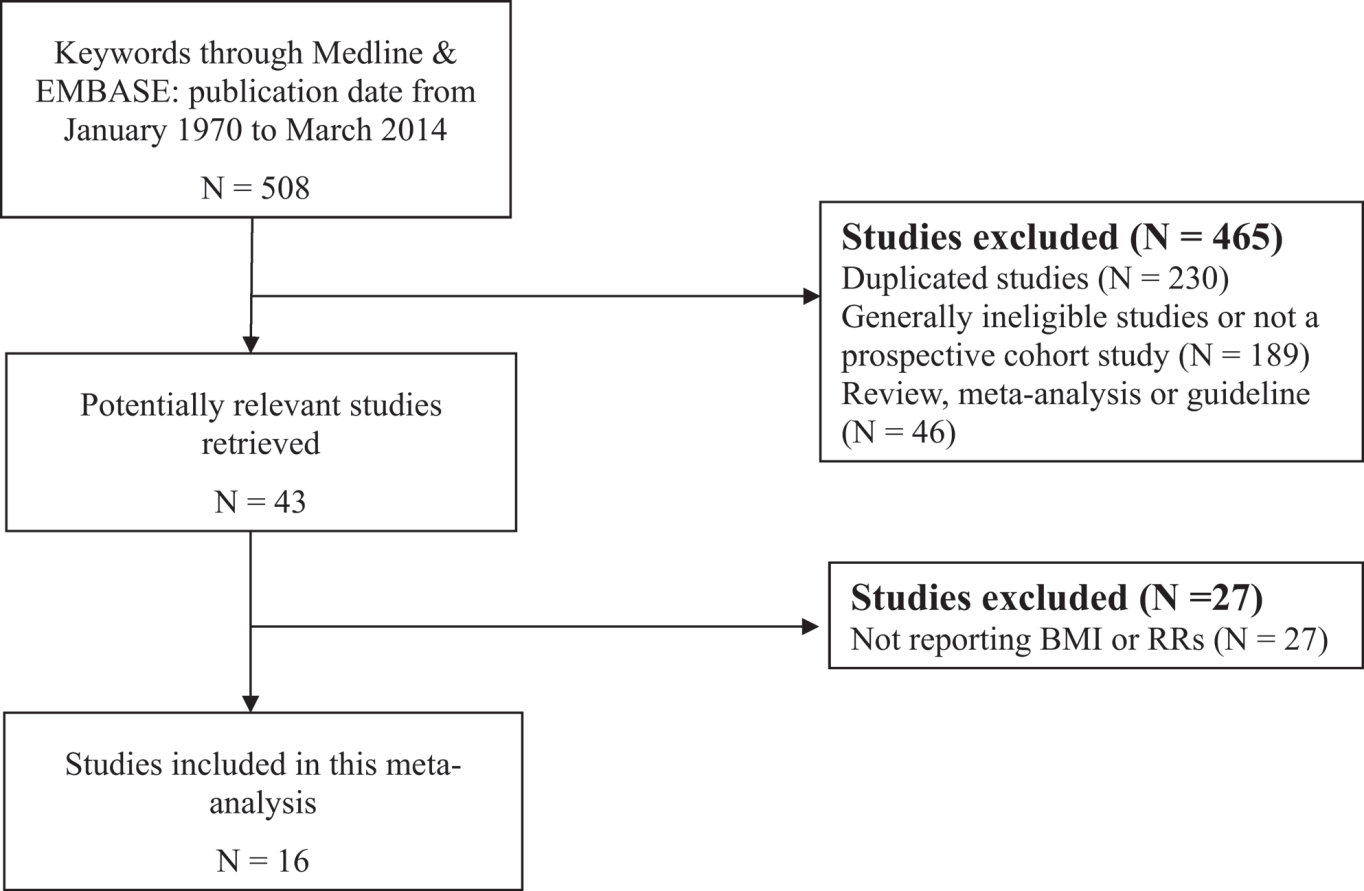
Flow diagram of the selection process for this meta-analysis. Details of the selection process are presented.

Using explicit inclusion and exclusion criteria, 16 articles were selected for this meta-analysis. Details of the selection process are presented (Fig. 1). The initial search yielded 508 articles. Among these, 465 were excluded because they were: duplicated studies, not reporting colorectal cancer-specific mortality or all-cause mortality, review or meta-analysis studies, or not a prospective cohort study. The remaining 43 potentially relevant studies were examined more closely and an additional 27 studies were excluded from analysis because of lack of information on BMI and the risk of mortality. Finally, 16 articles were selected for this meta-analysis based on these specific exclusion and inclusion criteria (Tables 1 and 2). The total sample size of patients with colorectal cancer in this meta-analysis was 58,917, encompassing both pre-diagnosis BMI and post-diagnosis BMI. The follow-up period ranged from 4.9 to 20 years (median: 9.9 years).

### Study Characteristics

Studies included in the meta-analyses used different BMI ranges for their underweight, reference overweight, and obese group. Among studies included in the meta-analysis, the range of BMI for underweight was < 18.5 kg/m^2^ (Dignam et al. [12], Prizment et al. [15], Baade et al. [7], Campbell et al. [16], Chin et al. [17], Kuiper et al. [11], Pelser et al. [18], and Schlesinger et al. [21]), < 20 kg/m^2^ (Meyerhardt et al. [8], Doria-Rose et al. [22], Sinicrope et al. [23], and Sinicrope et al. [13]), < 21 kg/m^2^ (Meyerhardt et al. [9] and Meyerhardt et al. [10]), and < 25 kg/m^2^ (Fedirko et al. [19] and Boyle et al. [20]).

The ranges used to define obesity categories also varied among studies. Four studies [9,12,13,23] of post-diagnosis BMI subdivided obesity as class I (30–34.9 kg/m^2^) and class II/III (BMI ≥ 35 kg/m^2^). To address these variances in categories, an additional meta-analysis was conducted to determine whether the diverse categories for the reference group influenced the results. The additional meta-analysis was conducted to compare results involving all studies without considering the differences in reference categories (18.5–24.9 kg/m^2^, 20–24.9 kg/m^2^, 21–24.9 kg/m^2^, and < 25 kg/m^2^) and obesity categories. This comparison analysis was conducted in most cases, but not in every case because the number of studies did not allow the completion of every possible meta-analysis. In none of the comparison was there evident statistically significant heterogeneity.

### Association between Pre-Diagnosis BMI and Mortality

**Fig 2:**
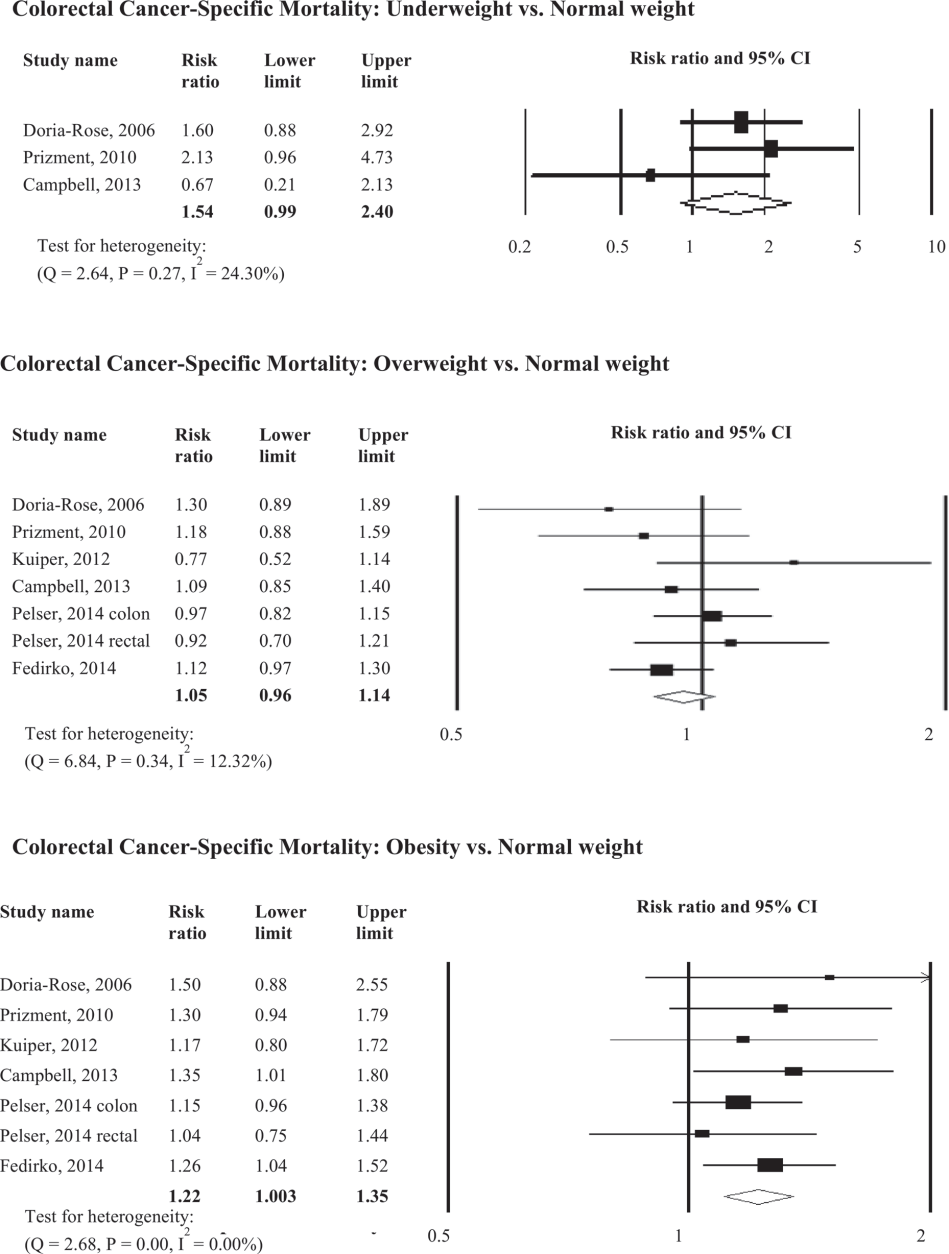
Relative Risks for the Association between Pre-diagnosis BMI and Colorectal Cancer-Specific and All-cause Mortality. Association between pre-diagnosis BMI and colorectal cancer-specific mortality and all-cause mortality.

Six studies reported an association between pre-diagnosis BMI and colorectal cancer-specific mortality and all-cause mortality (Fig. 2). Pre-diagnosis underweight was not significantly associated with colorectal cancer-specific mortality, but was significantly associated with all-cause mortality (RR: 1.63, 95% CI: 1.18–2.23, *p <* 0.01). Pre-diagnosis overweight was not associated with colorectal cancer-specific mortality and all-cause mortality. Pre-diagnosis obesity was significantly associated with increased colorectal cancer-specific mortality (RR: 1.22, 95% CI: 1.003–1.35, *p <* 0.01) and all-cause mortality (RR: 1.25, 95% CI: 1.14–1.36, *p <* 0.01). There was no evidence of publication bias in any analyses. Additionally, there was no apparent influence of unpublished data in any analyses using the trim and fill method.

### Association of Post-Diagnosis BMI with Mortality

**Fig 3:**
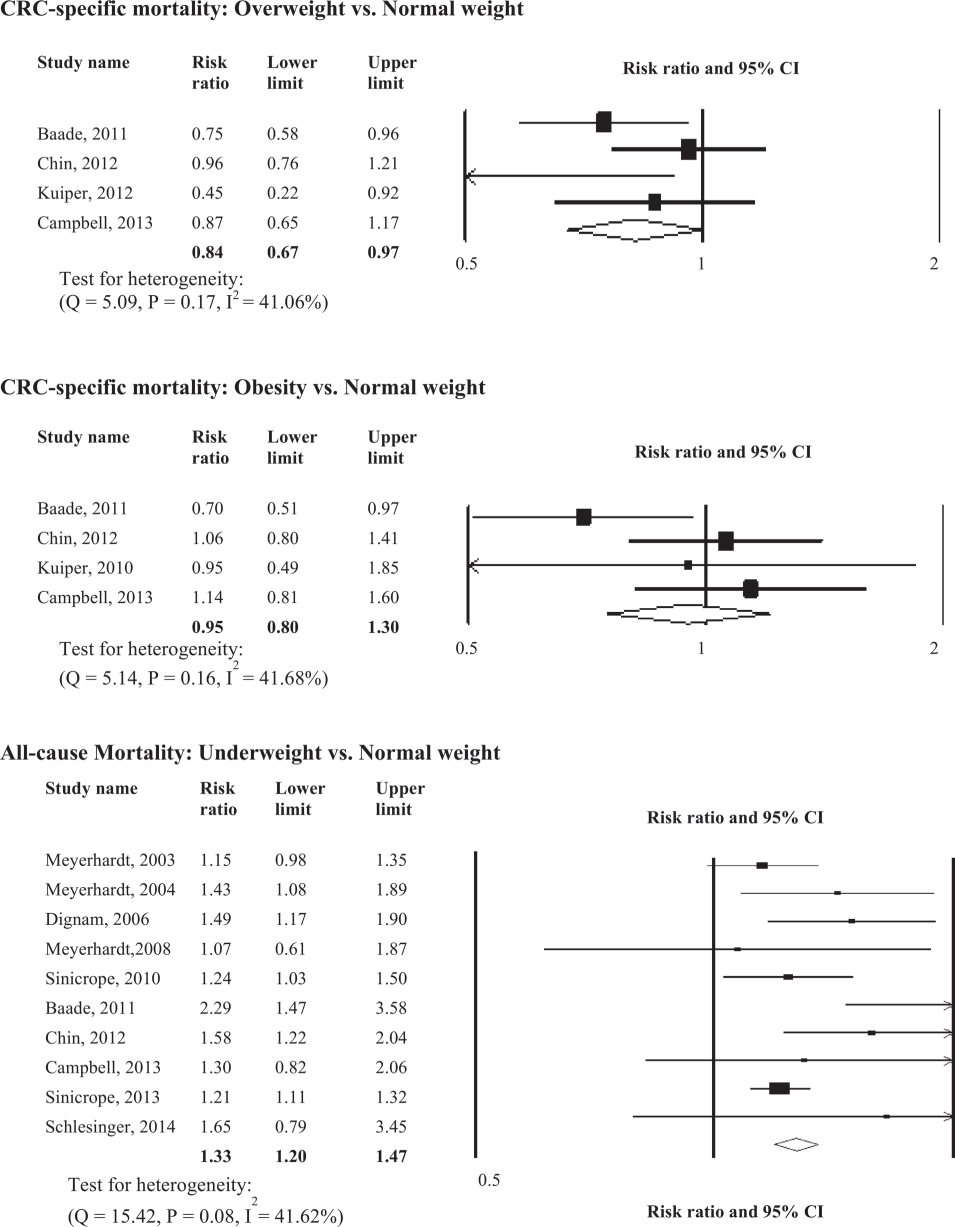
Relative Risks for the Association between Post-diagnosis BMI and Colorectal Cancer-specific and All-cause Mortality. Association between post-diagnosis BMI and colorectal cancer-specific mortality and all-cause mortality.

Twelve prospective cohort studies were included in this analysis (Fig. 3). Post-diagnosis underweight was associated with significantly increased all-cause mortality (RR: 1.33, 95% CI: 1.20–1.47, *p <* 0.01). Post-diagnosis overweight was associated with significantly improved colorectal cancer-specific mortality (RR: 0.84, 95% CI: 0.73–0.97, *p <* 0.05). Though post-diagnosis overweight was significantly but modestly associated with improved all-cause mortality (RR: 0.93, 95% CI: 0.86–0.997, *p <* 0.05). Since studies included in examining the association between post-diagnosis overweight and the risk of all-cause mortality were not homogeneous, we used random effect models and further analyzed the association between overweight and the risk of mortality according to different categories for normal BMI (18.5–24.9 kg/m^2^ vs. 20–24.9 kg/m^2^ vs. 21–24.9 kg/m^2^ vs. < 25 kg/m^2^). When we only included studies that used 20 or 21 kg/m^2^ as the lower limit for normal BMI range, the reduced risk of mortality observed among overweight participants no longer existed (RR: 0.98, 95% CI: 0.94–1.02, *p* = 0.32).

Post-diagnosis obesity was significantly associated with all-cause mortality (RR: 1.08, 95% CI: 1.03–1.13, *p <* 0.01) while no association was found between post-diagnosis obesity and colorectal cancer-specific mortality. We further analyzed the association between class II/III obesity (BMI > 35 kg/m^2^) and the risk of mortality and found significantly increased risk of all-cause mortality (RR: 1.13, 95% CI: 1.04–1.23, *p <* 0.01). There was neither evidence of publication bias in the analyses, nor apparent influence of unpublished data in any analyses using the trim and fill method.

### Association between Post-diagnosis BMI and Mortality in Subgroup Analysis: Gender

A subgroup analysis was performed to examine whether the association between post-diagnosis BMI and risk of mortality differed according to gender (there were too few cases of pre-diagnosis BMI for analysis. Post-diagnosis underweight was significantly associated with increased all-cause mortality in females (RR: 1.17, 95% CI: 1.08–1.28, *p* < 0.01) and in males (RR: 1.36, 95% CI: 1.02–1.82, *p* < 0.01). Post-diagnosis overweight was not significantly associated with increased all-cause mortality in females, but slightly associated with significantly reduced all-cause mortality in males (RR: 0.93, 95% CI: 0.88–0.98, *p* < 0.01). Post-diagnosis obesity was significantly associated with increased all-cause mortality in females (RR: 1.13, 95% CI: 1.05–1.21, *p* < 0.01) but only borderline statistically significant in males (RR: 1.05, 95% CI: 0.99–1.23, *p* = 0.13). No evidence of publication bias and no apparent influence of unpublished data using the trim and fill method was observed in any analyses.

### Association between Post-diagnosis BMI and Mortality in Subgroup Analysis: Site of Disease

Because there were insufficient studies to allow subgroup analysis for rectal cancer, this analysis only included colon cancer. Post-diagnosis underweight was significantly associated with all-cause mortality in patients with colon cancer (RR: 1.24, 95% CI: 1.16–1.32, *p* < 0.01). Post-diagnosis overweight was not significantly associated with all-cause mortality (RR: 1.04, 95% CI: 0.93–1.18, *p* = 0.48). Post-diagnosis obesity was associated with significantly increased all-cause mortality in colon cancer (RR: 1.09, 95% CI: 1.05–1.15, *p <* 0.01). The analyses did not show evidence of publication bias nor any apparent influence of unpublished data using the trim and fill method.

## Discussion

Apparently conflicting results on the association between BMI and the risk of mortality in individual studies may have resulted from the timing of BMI measurement, specifically whether it was before or after the cancer diagnosis. Therefore, we performed a meta-analysis of prospective cohort studies to distinguish the influence of pre- and post-diagnosis BMI on the risk of mortality in patients with colorectal cancer. Our analysis showed that pre-diagnosis underweight and obesity were associated with increased all-cause mortality and post-diagnosis underweight, class I obesity (BMI 30–34.9 kg/m^2^) and class II/III obesity (BMI ≥ 35 kg/m^2^) the increased risk of mortality.

We found that being obese before cancer diagnosis was associated with increased colorectal cancer-specific mortality as well as all-cause mortality and being obese post-diagnosis was associated with all-cause mortality in colorectal cancer survivors. In analyzing the impact of post-diagnosis BMI and the risk of mortality, it is important to understand the reason for the body weight change. There are two reasons for weight loss in colorectal cancer patients who completed standard adjuvant therapy: some patients intentionally lose weight through a healthier diet and exercise, whereas other patients experience weight loss due to progression of cancer or effects of treatment. Post-diagnosis BMI cannot distinguish between these reasons for weight loss, which might account for the lack of association between post-diagnosis BMI and the risk of mortality. However, in our meta-analysis of ten studies, we were able to find significant association between pre-diagnosis obesity and the risk of mortality. We further found higher relative risk of death due to all cause among patients with class II/III obesity (BMI ≥ 35 kg/m^2^). It is also important to understand that one of the main causes of death in obese colorectal cancer patients is cardiovascular disease rather than cancer recurrence [16], which may have contributed to overall mortality increase.

It is not clearly understood why those who were underweight before or after cancer diagnosis have increased risk of mortality. Considering that one of the symptoms of colorectal cancer is weight loss, patients with more advanced cancer at increased risk of mortality could experience more weight loss and are more likely to be underweight when cancer was diagnosed [8]. Studies in this area try to account and adjust for reverse causality by restricting the analysis with a lag period between measurement and time of event; however, such techniques likely cannot fully eliminate this effect. Furthermore, the reason for the increased risk of mortality among those who were underweight could be related to other diseases such as advanced type 2 diabetes [24], cardiac failure [25] and pulmonary diseases [32].

Our finding that being overweight was associated with a lower risk of mortality may not necessarily be causal but rather reflect that the reference group (“normal weight’) may include people who might have lost weight due to disease. Although the normal BMI category of 18.5–24.9 kg/m^2^ is recommended by the World Health Organization (WHO), some studies included in our meta-analysis used different classifications for normal BMI such as 20 or 21 kg/m^2^ as the lower cutoff. When we excluded studies that used BMI 18.5 kg/m^2^ as the lower cutoff for the normal range, we observed that post-diagnosis overweight was not significantly associated with reduced risk of mortality. A possible reason is that this exclusion reduces the number of people in the reference group with pre-existing disease or who have lost weight due to progressive colorectal cancer.

The biological mechanism that underlies the association between obesity and colorectal cancer mortality is unclear; but several studies have addressed possible mechanisms, mainly related to obesity-related hormonal changes. Obesity is associated with elevations in insulin, free insulin-like growth factors (IGFs), and adipocyte-derived factors that include leptin, TNF-alpha, IL-6, and reductions in adiponectin [26,27]. Many of these hormonal changes associated with obesity have been associated with increased incidence of colorectal cancer [14,28–30], though because of their inter-relations, it has been difficult to conclude which ones are causal. Studies on these in relation to survival have been scarce, though Wolpin et al. [31] reported that a higher pre-diagnostic level of C-peptide, which reflects insulin secretion, was associated with increased colorectal cancer mortality in patients with colorectal cancer (HR: 1.87, 95% CI, 1.04–0.36, *p* = 0.03). Since physical activity, exercise and reducing body fat have positively influence on these factors, healthier lifestyle including exercise and maintaining health body weight should be recommended to improve prognosis of colorectal cancer patients [33]. Several recent meta-analyses have reported associations between vegetable, aspirin, and supplements, with colorectal cancer risk or mortality, but further study needs to be conducted to find a consistent association [36,37].

There are strengths and limitations of the current study. First, we only included prospective cohort studies that would be less susceptible to selection and recall biases. In addition, we have performed meta-analysis separately addressed BMI before and after diagnosis to obtain association with all-cause and colorectal cancer-specific mortality. Combining studies allowed us greater power to observe associations.

We also have several limitations. The major limitation is the possibility of reverse causation, which probably precludes us identifying a pure group of healthy normal weight persons. Thus, we likely underestimated the true impact of obesity on colorectal prognosis because progressing cancers typically cause weight loss rather than weight gain. A second limitation is that pre-diagnosis and post-diagnosis BMI are correlated with each other and therefore we cannot precisely say when of obesity is acting. Nonetheless, since pre-diagnosis BMI is a major determinant of post-diagnosis BMI, it is desirable to maintain normal weight throughout life rather than relying on weight loss after diagnosis. Another limitation of the study is that several studies in this meta-analysis used self-reported BMI, though limitation is probably minor as self-reported BMI was shown to be highly correlated with measured BMI [34]. Finally, BMI alone may not provide adequate information to classify body fat and lean mass and fat distribution, and these characteristics can vary significantly based on gender, age, ethnicity, and geographic region [4,14].

There are additional considerations that might influence the association between body mass index and colorectal cancer mortality in both pre-diagnosis and post-diagnosis. These factors include disease severity at diagnosis, prognosis factors, and extend to discern how findings may differ across various population groups. The current meta-analysis used RRs that were adjusted for those factors, including disease severity at diagnosis, prognosis factors, sex, age, etc. However, the exact same factors were no uniformly applied throughout all studies. Future meta-analysis studies should conduct more stratified analyses that examine a variety of populations and examine the effects of other adjustment factors. Additionally, B-Catenin status, which plays an important role in carcinogenesis of colorectal cancer, also needs to be studied to better understand the mechanism related to adiposity, physical activity, and colorectal cancer [38,39].

In conclusion, the findings of this meta-analysis suggest that both pre- and post-diagnosis underweight and obesity are associated with increased risk of mortality. Maintaining a normal body weight should be considered by all individuals including colorectal cancer patients. Intervention studies on the impact of weight control on the risk of mortality in colorectal cancer patients are needed.

## Supporting Information

S1 Table Prisma 2009 checklist. This study used PRISMA guidance to help improve reporting quality of this meta-analysis study.
(PDF)

## References

[1] Ferlay J, Shin HR, Bray F, Forman D, Mathers C, Parkin DM (2010) Estimates of worldwide burden of cancer in 2008: GLOBOCAN 2008. Int J Cancer 127: 2893–2917. 10.1002/ijc.25516 21351269

[2] Liberati A, Altman DG, Tetzlaff J, Mulrow C, Gotzsche PC, Ioannidis JP, et al (2009) The PRISMA statement for reporting systematic reviews and meta-analyses of studies that evaluate health care interventions: explanation and elaboration. J Clin Epidemiol 62:e1–e34. 10.1016/j.jclinepi.2009.06.006 19631507

[3] Harriss DJ, Atkinson G, George K, Cable NT, Reilly T, Haboubi N, et al (2009) Lifestyle factors and colorectal cancer risk (1): systematic review and meta-analysis of associations with body mass index. Colorectal Dis 11: 547–563. 10.1111/j.1463-1318.2009.01766.x 19207714

[4] Wells GA, Shea B, O’Connell D, Peterson J, Welch V, Tugwell P (2012) The Newcastle-Ottawa Scale (NOS) for assessing the quality of nonrandomised studies in metaanalyses. Presented at the 3rd Symposium on Systematic Reviews Beyond the Basics, July 3–5, 2000, Oxford, UK.

[5] Okabayashi K, Ashrafian H, Hasegawa H, Yoo JH, Patel VM, Harling L, et al (2012) Body mass index category as a risk factor for colorectal adenomas: a systematic review and meta-analysis. Am J Gastroenterol 107: 1175–1185; quiz 1186. 10.1038/ajg.2012.180 22733302

[6] Ma Y, Yang Y, Wang F, Zhang P, Shi C, Zou Y, et al (2013) Obesity and risk of colorectal cancer: a systematic review of prospective studies. PLoS One 8: e53916 10.1371/journal.pone.0053916 23349764 PMC3547959

[7] Baade PD, Meng X, Youl PH, Aitken JF, Dunn J, Chambers SK (2011) The impact of body mass index and physical activity on mortality among patients with colorectal cancer in Queensland, Australia. Cancer Epidemiol Biomarkers Prev 20: 1410–1420. 10.1158/1055-9965.EPI-11-0079 21558496

[8] Meyerhardt JA, Tepper JE, Niedzwiecki D, Hollis DR, McCollum AD, Brady D, et al (2004) Impact of body mass index on outcomes and treatment-related toxicity in patients with stage II and III rectal cancer: findings from Intergroup Trial 0114. J Clin Oncol 22: 648–657. 14966087 10.1200/JCO.2004.07.121

[9] Meyerhardt JA, Niedzwiecki D, Hollis D, Saltz LB, Mayer RJ, Nelson H, et al (2008) Impact of body mass index and weight change after treatment on cancer recurrence and survival in patients with stage III colon cancer: findings from Cancer and Leukemia Group B 89803. J Clin Oncol 26: 4109–4115. 10.1200/JCO.2007.15.6687 18757324 PMC2654367

[10] Meyerhardt JA, Catalano PJ, Haller DG, Mayer RJ, Benson AB 3rd, Macdonald JS, et al (2003) Influence of body mass index on outcomes and treatment-related toxicity in patients with colon carcinoma. Cancer 98: 484–495. 12879464 10.1002/cncr.11544

[11] Kuiper JG, Phipps AI, Neuhouser ML, Chlebowski RT, Thomson CA, Irwin ML, et al (2012) Recreational physical activity, body mass index, and survival in women with colorectal cancer. Cancer Causes Control 23: 1939–1948. 10.1007/s10552-012-0071-2 23053793 PMC3499635

[12] Dignam JJ, Polite BN, Yothers G, Raich P, Colangelo L, O’Connell MJ, et al (2006) Body mass index and outcomes in patients who receive adjuvant chemotherapy for colon cancer. J Natl Cancer Inst 98: 1647–1654. 17105987 10.1093/jnci/djj442

[13] Sinicrope FA, Foster NR, Yothers G, Benson A, Seitz JF, Labianca R, et al (2013) Body mass index at diagnosis and survival among colon cancer patients enrolled in clinical trials of adjuvant chemotherapy. Cancer 119: 1528–1536. 10.1002/cncr.27938 23310947 PMC3769640

[14] Larsson SC, Orsini N, Wolk A (2005) Diabetes mellitus and risk of colorectal cancer: a meta-analysis. J Natl Cancer Inst 97: 1679–1687. 16288121 10.1093/jnci/dji375

[15] Prizment AE, Flood A, Anderson KE, Folsom AR (2010) Survival of women with colon cancer in relation to precancer anthropometric characteristics: the Iowa Women's Health Study. Cancer Epidemiol Biomarkers Prev 19: 2229–2237. 10.1158/1055-9965.EPI-10-0522 20826830 PMC2945249

[16] Campbell PT, Newton CC, Dehal AN, Jacobs EJ, Patel AV, Gapstur SM (2012) Impact of body mass index on survival after colorectal cancer diagnosis: the Cancer Prevention Study-II Nutrition Cohort. J Clin Oncol 30: 42–52. 10.1200/JCO.2011.38.0287 22124093

[17] Chin CC, Kuo YH, Yeh CY, Chen JS, Tang R, Changchien C-R, et al (2012) Role of body mass index in colon cancer patients in Taiwan. World J Gastroenterol 18: 4191–4198. 10.3748/wjg.v18.i31.4191 22919253 PMC3422801

[18] Pelser C, Arem H, Pfeiffer RM, Elena JW, Alfano CM, Hollenbeck AR, et al (2014) Prediagnostic lifestyle factors and survival after colon and rectal cancer diagnosis in the National Institutes of Health (NIH)-AARP Diet and Health Study. Cancer. 120 (10):1540–1547. 10.1002/cncr.28573 24591061 PMC4151292

[19] Fedirko V, Romieu I, Aleksandrova K, Pischon T, Trichopoulos D, Pischon T, et al (2014) Pre-diagnostic anthropometry and survival after colorectal cancer diagnosis in Western European populations. Int J Cancer 135: 1949–1960. 10.1002/ijc.28841 24623514

[20] Boyle T, Fritschi L, Platell C, Heyworth J (2013) Lifestyle factors associated with survival after colorectal cancer diagnosis. Br J Cancer 109: 814–822. 10.1038/bjc.2013.310 23787918 PMC3738138

[21] Schlesinger S, Siegert S, Koch M, Walter J, Heits N, Hinz S, et al (2014) Postdiagnosis body mass index and risk of mortality in colorectal cancer survivors: a prospective study and meta-analysis. Cancer Causes Control 25: 1407–1418. 10.1007/s10552-014-0435-x 25037235

[22] Doria-Rose VP, Newcomb PA, Morimoto LM, Hampton JM, Trentham-Dietz A (2006) Body mass index and the risk of death following the diagnosis of colorectal cancer in postmenopausal women (United States). Cancer Causes Control 17: 63–70. 16411054 10.1007/s10552-005-0360-0

[23] Sinicrope FA, Foster NR, Sargent DJ, O'Connell MJ, Rankin C (2010) Obesity is an independent prognostic variable in colon cancer survivors. Clin Cancer Res 16: 1884–1893. 10.1158/1078-0432.CCR-09-2636 20215553 PMC2948494

[24] Sairenchi T, Iso H, Irie F, Fukasawa N, Ota H, Muto T (2008) Underweight as a predictor of diabetes in older adults: a large cohort study. Diabetes Care 31:583–4. 18071003 10.2337/dc07-1390

[25] Kenchaiah S, Pocock SJ, Wang D, Finn PV, Zornoff LAM, Skali H, et al (2007) Body mass index and prognosis in patients with chronic heart failure: insights from the Candesartan in Heart failure: Assessment of Assessment of Reduction in Mortality and morbidity (CHARM) program. Circulation 116:627–36. 17638930 10.1161/CIRCULATIONAHA.106.679779

[26] Renehan AG, Roberts DL, Dive C (2008) Obesity and cancer: pathophysiological and biological mechanisms. Arch Physiol Biochem 114: 71–83. 10.1080/13813450801954303 18465361

[27] Renehan AG, Frystyk J, Flyvbjerg A (2006) Obesity and cancer risk: the role of the insulin-IGF axis. Trends Endocrinol Metab 17: 328–336. 16956771 10.1016/j.tem.2006.08.006

[28] Stattin P, Lukanova A, Biessy C, Soderberg S, Palmqvist R, Kaaks R, et al (2004) Obesity and colon cancer: does leptin provide a link? Int J Cancer 109: 149–152. 14735482 10.1002/ijc.11668

[29] Fasshauer M, Paschke R (2003) Regulation of adipocytokines and insulin resistance. Diabetologia 46: 1594–1603. 14605806 10.1007/s00125-003-1228-z

[30] Chung YW, Han DS, Park YK, Son BK, Paik CH, Lee HL, et al (2006) Association of obesity, serum glucose and lipids with the risk of advanced colorectal adenoma and cancer: a case-control study in Korea. Dig Liver Dis 38: 668–672. 16790371 10.1016/j.dld.2006.05.014

[31] Wolpin BM, Meyerhardt JA, Chan AT, Ng K, Chan JA, Wu K, et al (2009) Insulin, the insulin-like growth factor axis, and mortality in patients with nonmetastatic colorectal cancer. Journal of clinical oncology: official journal of the American Society of Clinical Oncology 27: 176–185. 19064975 10.1200/JCO.2008.17.9945 PMC2645084

[32] Cao C, Wang R, Wang J, Bunjhoo H, Xu Y, Xiong W (2012) Body mass index and mortality in chronic obstructive pulmonary disease: a meta-analysis. PLoS One 7:e43892 10.1371/journal.pone.0043892 22937118 PMC3427325

[33] Jeon JY, Jeong DH, Park MG, Lee J-W, Chu SH, Park J-H, et al (2013) Impact of diabetes on oncologic outcome of colorectal cancer patients: colon vs. rectal cancer. PLoS One 8:e55196 10.1371/journal.pone.0055196 23405123 PMC3566217

[34] Palta M, Prineas RJ, Berman R, Hannan P (1982) Comparison of self-reported and measured height and weight. Am J Epidemiol 115: 223–230. 7058781 10.1093/oxfordjournals.aje.a113294

[35] Parkin E, O'Reilly DA, Sherlock DJ, Manoharan P, Renehan AG (2014) Excess adiposity and survival in patients with colorectal cancer: a systematic review. Obes Rev 15: 434–451. 10.1111/obr.12140 24433336

[36] Li P, Wu H, Zhang H, Shi Y, Xu J, Ye Y, et al. (2014) Aspirin use after diagnosis but not prediagnosis improves established colorectal cancer survival: a meta-analysis. Gut. 10.1136/gutjnl-2014-308260 25239119

[37] Zhu B, Zou L, Qi L, Zhong R, Miao X (2014) Allium Vegetables and Garlic Supplements Do Not Reduce Risk of Colorectal Cancer, Based on Meta-analysis of Prospective Studies. Clinical gastroenterology and hepatology: the official clinical practice journal of the American Gastroenterological Association 12 (12):1991–2001 e1994. 10.1016/j.cgh.2014.03.019 24681077

[38] Morikawa T, Kuchiba A, Yamauchi M, Meyerhardt JA, Shima K, Nosho K, et al (2011) Association of CTNNB1 (beta-catenin) alterations, body mass index, and physical activity with survival in patients with colorectal cancer. Jama 305 (16):1685–1694. 10.1001/jama.2011.513 21521850 PMC3087286

[39] Morikawa T, Kuchiba A, Lochhead P, Nishihara R, Yamauchi M, Imamura Y, et al (2013) Prospective analysis of body mass index, physical activity, and colorectal cancer risk associated with beta-catenin (CTNNB1) status. Cancer research 73 (5):1600–1610. 10.1158/0008-5472.CAN-12-2276 23442321 PMC3594537

