# Correction: Association between Body Mass Index and Prognosis of Colorectal Cancer: A Meta-Analysis of Prospective Cohort Studies

**DOI:** 10.1371/journal.pone.0147456

**Published:** 2016-01-14

**Authors:** The PLOS ONE Staff

Fig 2, “Relative Risks for the Association between Pre-diagnosis BMI and Colorectal Cancer-Specific and All-cause Mortality,” has been truncated. Please view Fig 2 here. The publisher apologizes for the error.

**Fig 2:**
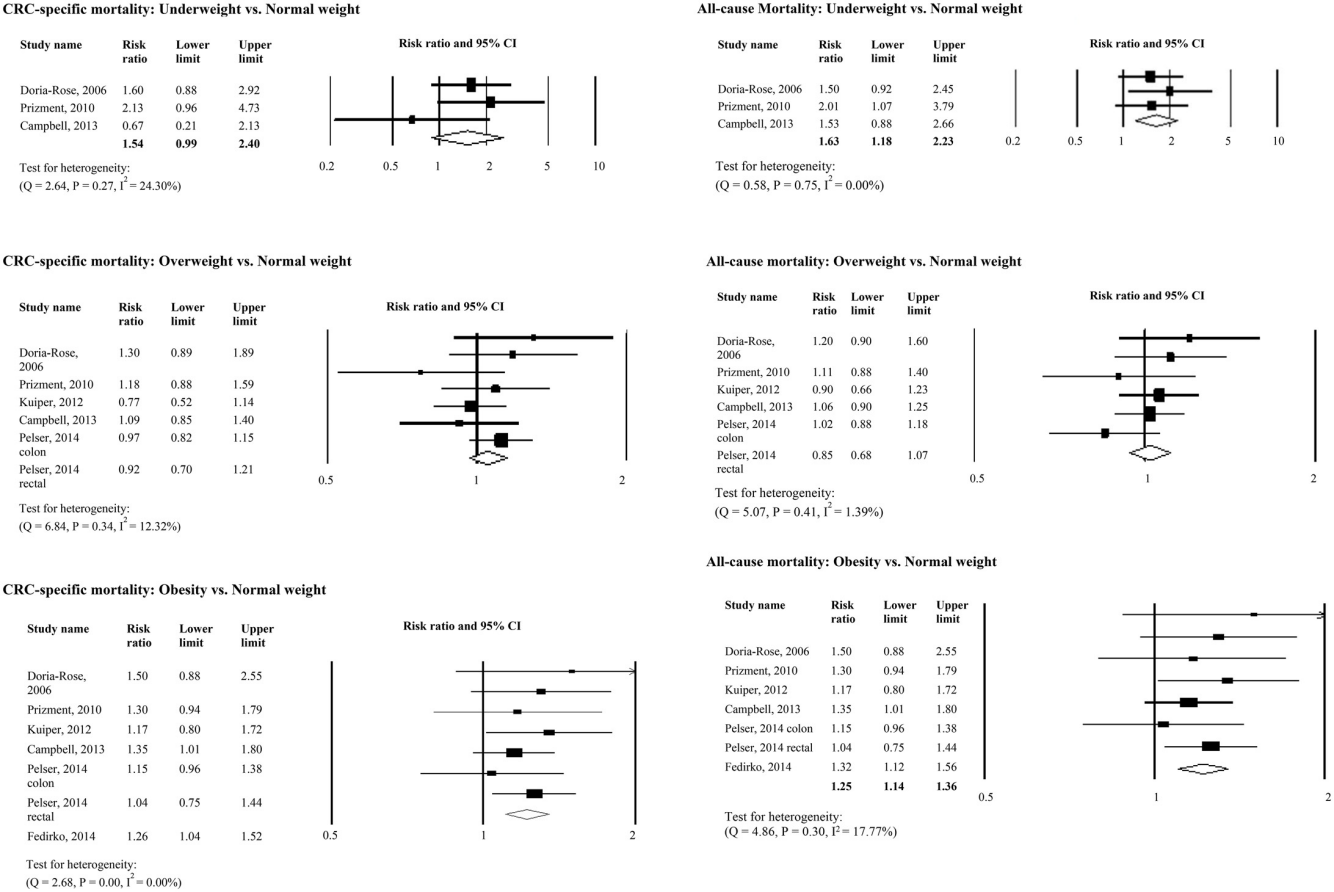
Relative Risks for the Association between Pre-diagnosis BMI and Colorectal Cancer-Specific and All-cause Mortality. Association between pre-diagnosis BMI and colorectal cancer-specific mortality and all-cause mortality.

Fig 3, “Relative Risks for the Association between Post-diagnosis BMI and Colorectal Cancer-specific and All-cause Mortality,” has been truncated. Please view Fig 3 here. The publisher apologizes for the error.

**Fig 3:**
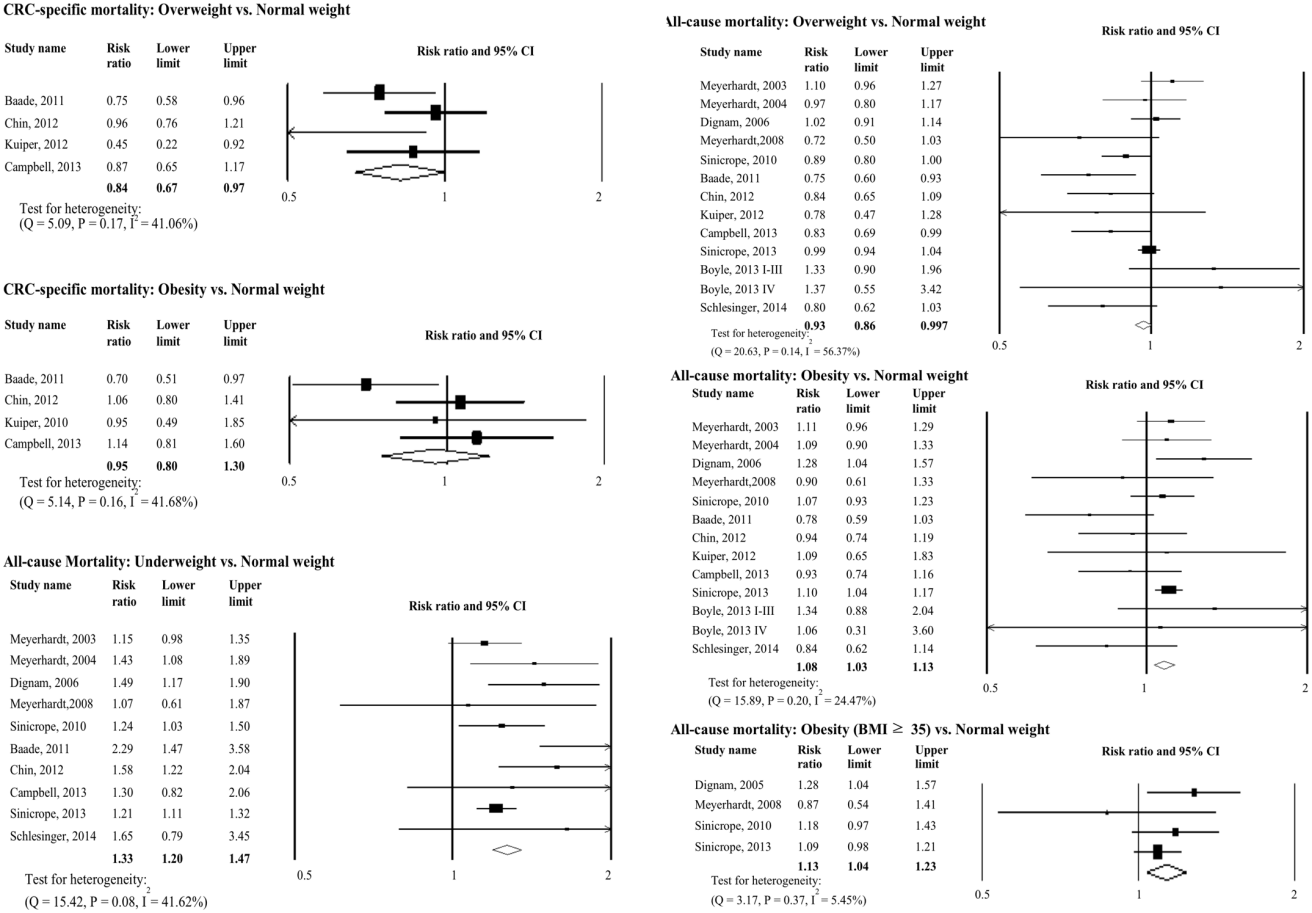
Relative Risks for the Association between Post-diagnosis BMI and Colorectal Cancer-specific and All-cause Mortality. Association between post-diagnosis BMI and colorectal cancer-specific mortality and all-cause mortality.
